# Risk of COVID-19 importation to the Pacific islands through global air travel

**DOI:** 10.1017/S0950268820000710

**Published:** 2020-03-23

**Authors:** A. T. Craig, A. E. Heywood, J. Hall

**Affiliations:** School of Public Health and Community Medicine, University of New South Wales, Sydney, Australia

**Keywords:** Coronavirus, COVID-19, Pacific islands, surveillance, travel

## Abstract

On 30 January 2020, WHO declared coronavirus (COVID-19) a global public health emergency. As of 12 March 2020, 125 048 confirmed COVID-19 cases in 118 countries had been reported. On 12 March 2020, the first case in the Pacific islands was reported in French Polynesia; no other Pacific island country or territory has reported cases. The purpose of our analysis is to show how travellers may introduce COVID-19 into the Pacific islands and discuss the role robust health systems play in protecting health and reducing transmission risk. We analyse travel and Global Health Security Index data using a scoring tool to produce quantitative estimates of COVID-19 importation risk, by departing and arriving country. Our analysis indicates that, as of 12 March 2020, the highest risk air routes by which COVID-19 may be imported into the Pacific islands are from east Asian countries (specifically, China, Korea and Japan) to north Pacific airports (likely Guam, Commonwealth of the Northern Mariana Islands or, to a less extent, Palau); or from China, Japan, Singapore, the United States of America or France to south Pacific ports (likely, Fiji, Papua New Guinea, French Polynesia or New Caledonia). Other importation routes include from other east Asian countries to Guam, and from Australia, New Zealand and other European countries to the south Pacific. The tool provides a useful method for assessing COVID-19 importation risk and may be useful in other settings.

## Introduction

On 31 December 2019, the Chinese government alerted WHO to several severely ill cases of pneumonia; and on 7 January 2020, announced that a novel coronavirus (later named Sudden Acute Respiratory Syndrome – Coronavirus-2 (SARS-CoV-2)) was the cause of the illness [1]. On 30 January 2020, following a worsening situation in China and the emergence of coronavirus (COVID-19) cases in 19 other countries, WHO declared the outbreak a ‘public health emergency of international concern’ under the International Health Regulations (2005) (IHR (2005)) [2, 3].

At the time of writing (13 March 2020), 118 countries, territories and areas had reported COVID-19 cases with local transmission in 72 [4]. On 12 March 2020, authorities in French Polynesia announced the importation of a COVID-19 case [5], the first case to be detected in the Pacific islands.

**Fig. 1.**
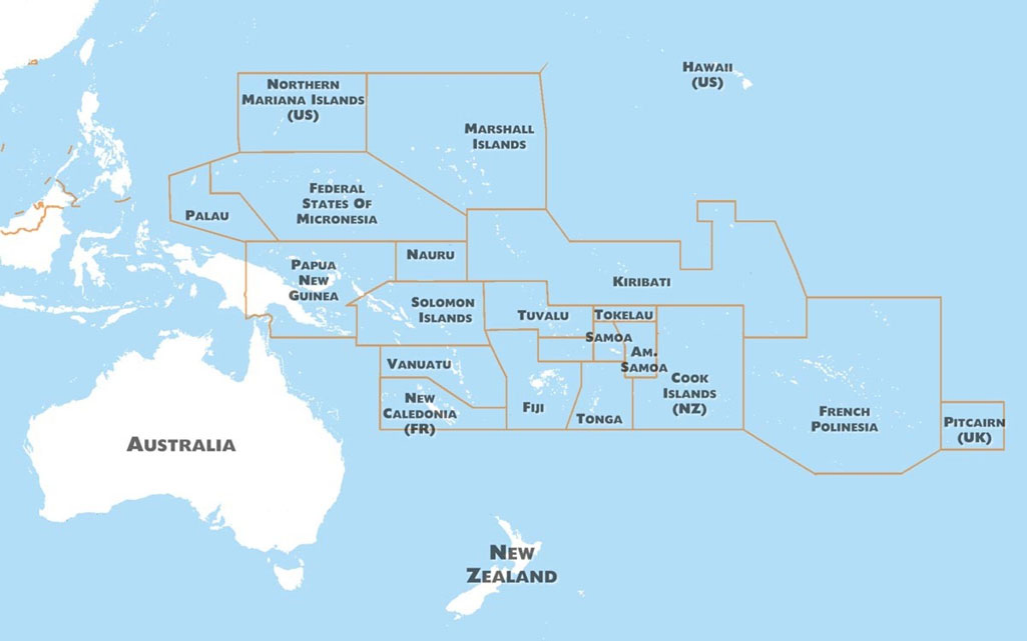
Map of the Pacific island region.

The Pacific region covers one-third of the earth and is home to approximately 12 million people (excluding Australia and New Zealand) (Fig. 1). Of these, 8.3 million reside in Papua New Guinea (PNG), with the remainder dispersed over the many thousands of islands and atolls that make up the other 21 Pacific island countries and territories (PICTs). Several of the world's smallest, least developed and most isolated populations are in the Pacific. Fourteen Pacific island countries are States Parties to the IHR (2005), and seven are territories or administrative areas for which IHR (2005) responsibilities are delegated to their metropolitan country [6].

Health system strengthening is a key development need for many PICTs, and, given the weak state of some islands' health sectors [6–10] there are concerns that authorities will struggle to respond, should COVID-19 be detected within their borders [11, 12].

History has shown that global public health emergencies can have an extraordinarily high impact on the PICTs. For example, the 1918 influenza pandemic was estimated to have resulted in a mortality rate of as high as 22% in some countries [13], and – more recently – outbreaks of arboviral diseases [14, 15] and measles [16] have had devastating effects on Pacific populations.

While relatively isolated, the interconnectedness of air travel networks shows that PICTs are still vulnerable to global disease threats. The purpose of this analysis is to show how travellers may introduce COVID-19 into the PICTs and discuss the role robust health systems play in health protection and transmission risk reduction.

Several recently published papers predict the international spread of COVID-19 through global air travel [17–19]. We build on the methods presented in these papers to produce analysis specific to the Pacific island regions. Our analysis considers importation risk from all countries affected by COVID-19 as of 12 March 2020 and emphasises the important role robust health systems play in mitigating risk.

## Methods

We analysed International Air Traffic Association (IATA) data for all inbound international transits to PICT airports for four randomly selected months (January, April, July and November) in 2019 to estimate passenger transit volume by route, and national GHS Index [20] scores as a measure of affected countries' capacity to detect and respond to COVID-19. Travel data used was for single ticketed international passenger transits on commercial airlines (i.e. these data do not include military-related international travel). Travel where passengers ‘broke’ their journeys (i.e. stop in a country on-route) is recorded in the dataset as two separate journeys. We exclude travel routes with less than 1000 transits per year from the analysis. The GHS Index is based on an analysis of open-source information about 140 elements, organised into six categories, 34 indicators and 85 sub-indicators. The six categories are (i) ‘prevention’ (i.e. prevention of the emergence or release of pathogens); (ii) ‘detection and reporting’ (i.e. early detection and reporting for epidemics of potential international concern); (iii) ‘rapid response’ (i.e. rapid response to and mitigation of the spread of an epidemic); (iv) ‘health system’ (sufficient and robust health system to treat the sick and protect health workers); (v) ‘compliance with international norms’ (i.e. commitments to improving national capacity, financing plans to address gaps and adhering to global norms) and (vi) ‘risk environment’ (i.e. overall risk environment and country vulnerability to biological threats) [21].

We analysed these datasets using a simple scoring tool we developed to produce a stratified estimate of relative risk of COVID-19 importation to PICTs. The tool rates four risk elements, by COVID-19 affected country. These are (i) the number of confirmed COVID-19 cases; (ii) evidence of local transmission; (iii) the overall health security preparedness rank (i.e. level of preparedness), as reported in the GHS Index and (iv) the passenger volume to a PICT airport. For each risk element, we assign a score out of three. For the ‘number of confirmed cases’ element we assign a score of ‘1’ if WHO reported less than 20 confirmed COVID-19 cases, ‘2’ if there was between 20 and 150 reported cases and ‘3’ if there were more than 150 reported cases. For the evidence of local transmission element, we assigned a score of ‘1’ if WHO reported ‘imported cases only’ or ‘under investigation’ and ‘3’ if WHO reported ‘local transmission’. For the ‘overall health security preparedness rank’ element, we assign a score of ‘1’ if the country was categorised (in relation to all other countries) as ‘most prepared’ in the GHS Index, ‘2’ if categories as ‘more prepared’, and ‘3’ if categorised as ‘least prepared’. While for the ‘passenger volume’ element we assign a score of ‘1’ if the estimated number of passengers arriving from a country to a PICT airport was less than 10 000 per annum, ‘2’ if it was between 10 000 and 100 000 and ‘3’ if it was more than 100 000. We then added the risk element scores and if the sum of the scores was less than 7, we describe the risk of COVID-19 importation from the respective country to be in the ‘lowest’ likelihood category, if the sum of scores was between 7 and 9, we describe the risk as being in the ‘moderate’ likelihood category and if the sum of scores was greater than 9, we describe the risk as being in the ‘highest’ category.

We consider the PICTs in two subregions (PICTs located north of the equator (i.e. in the North Pacific) and those located south of the equator (i.e. in the South Pacific)) as there are distinctly different travel routes and patterns to and between each. The PICTs in the north Pacific are the CNMI, Guam, Palau, the Federated States of Micronesia (FSM) and the Marshall Islands; and the PICTs in the south Pacific are PNG, Solomon Islands, Vanuatu, New Caledonia, Fiji, Nauru, Tuvalu, Kiribati, Tonga, Samoa, American Samoa, Wallis and Futuna, French Polynesia, Niue, Cook Islands, Tokelau and Pitcairn Island.

## Results

### Travel into the Pacific islands for COVID-19 affected countries

**Fig. 2.**
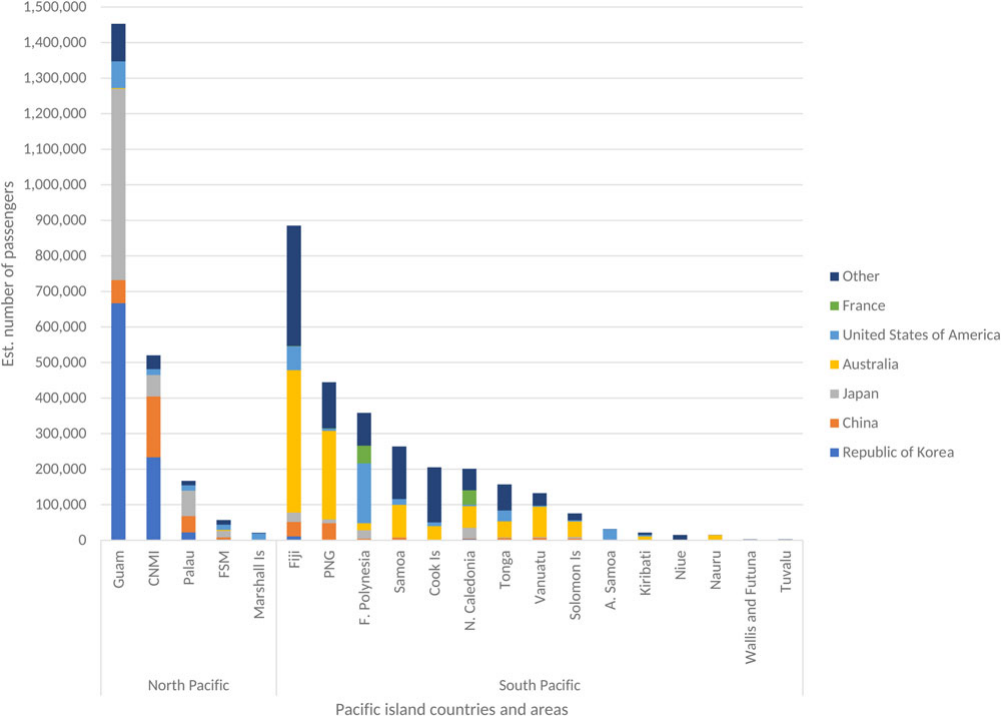
Estimated number of passengers from COVID-19 affected countries (as of 2 March 2020) to the Pacific islands, by departing country.

We estimate that approximately 5 million passengers/year arrive at a PICT airport from a country that (as of 12 March 2020) has reported COVID-19 cases. Most inbound travellers' final destination of ticketed route was Guam (28.9% of all PICT arrivals from COVID-19 affected countries), Fiji (17.6%), CNMI (10.3%), PNG (8.8%) or French Polynesia (7.1%) (Fig. 2).

There is a clear north/south travel divide in the Pacific with less than 5% of inter-PICT transits crossing the equator. The five most frequented inbound routes from countries affected by COVID-19 (as of 12 March 2020) to PICTs located in the north Pacific were Korea to Guam (approximately 667 000 passengers/year) and CNMI (approximately 223 000 passengers/year); Japan to Guam (approximately 538 000 passengers/year) and Palau (approximately 72 000 passengers/year) and the United States of America (USA) to Guam (approximately 75 500 passengers/year). To PICTs located in the south Pacific, the most frequented routes were Australia to Fiji (approximately 401 000 passengers/year) and PNG (approximately 250 000 passengers/year); New Zealand to Fiji (approximately 261 000 passengers/year) and Samoa (approximately 139 000 passengers/year); and the USA to French Polynesia (approximately 169 000 passengers/year) (Fig. 2). Guam serves as the main transit point to other PICTs located north of the equator, while Fiji provides the same function to PICTs located south of the equator. Supplementary Table S1 provides estimated passenger travel volume between COVID-19 affected countries (as of 12 March 2020) and PICTs.

### Health system strength and capacity to respond

Our analysis shows that countries currently affected by COVID-19 tend to be, relatively to all countries, well prepared and able to detect and respond to outbreaks. For example, 68 of the 112 COVID-19 affected countries (60.7%) (note: the six COVID-19 affected territories and areas are not included) have an overall GHS Index score greater than the global mean. This is in stark contrast to the situation in the PICTs where all countries for which GHS Index data is reported fall within the ‘least prepared’ stratum of the measure.

### Route and risk of COVID-19 importation to the Pacific islands

While correlation between importation risk, international travel and the spatial distribution of COVID-19 cases is intuitive, our findings suggest that descriptive analysis is helpful for identification, assessment and communicating probability. Our analysis indicates that as of 12 March 2020, the highest risk air routes by which COVID-19 may be imported into the Pacific islands are from east Asian countries (specifically, China, Korea and Japan) to north Pacific airports (likely Guam, CNMI or, to a less extent, Palau); or from China, Japan, Singapore, USA or France to south Pacific ports (likely, Fiji, PNG, French Polynesia or New Caledonia) (Table 1; Supplementary Table S1). In the event of continued global spread of COVID-19, additional importation routes include from other east Asian countries to Guam, and from Australia, New Zealand and other European countries to Fiji, Samoa, French Polynesia, New Caledonia and the smaller PICTs.

**Table 1.** Highest risk international air routes by which COVID-19 may be imported into the Pacific islands (as of 12 March 2020), by COVID-19 affected countries.

North Pacific		South Pacific	
From	To	From	To
China^a^	CNMI, Guam, Palau	China^a^	PNG, Fiji
Republic of Korea	Guam, CNMI, Palau	Japan	N. Caledonia, Fiji, F. Polynesia
Japan	Guam, Palau, CNMI	Singapore	PNG, Fiji
		France	F. Polynesia, N. Caledonia
		USA	F. Polynesia

CNMI, Commonwealth of the Northern Mariana Islands; F. Polynesia, French Polynesia; N. Caledonia, New Caledonia; PNG, Papua New Guinea; USA, United States of America.

a Including Hong Kong, Macau and Taiwan.

## Discussion

We present a simple method by which open-source data may be used to estimate the risk of COVID-19 importation to currently unaffected countries. We apply the tool to the Pacific context and identify the international commercial air traffic routes that pose the greatest risk of case importation is (at the time of writing) from Asia, Europe or USA with the most likely importation route through the busiest Pacific ports of Guam or CNMI in the north Pacific, or Fiji, PNG or French Polynesia in the south Pacific.

As many PICTs struggle to deliver even the most basic public health services, international assistance to help prepare for and respond to COVID-19 is essential. Efforts should focus on enhancing capacity to provide care for severely ill cases, including ensuring access to medical oxygen, ventilators and other vital equipment; ensuring equipment is available; that protocols are in place to prevent and manage hospital-acquired infections; and that the workforce is trained and equipped to stay safe; surveillance is adequate to identify and track cases in the initial stages of an outbreak, and monitor the temporo-spatial distribution and severity of illness if the disease spreads; that laboratories are resourced to test samples; that measures are in place at airports to detect and treat ill travellers and that risk communication mechanisms are in place to ensure the public has access to the correct information and advice.

The effort made to strengthening PICTs' capacity to respond to COVID-19 offers an opportunity to address underlying health system barriers that have inhibited PICTs' ability to independently respond to public health emergencies. Hence, where possible, development support should be delivered within a health system strengthening frame [22]; that is, actions that foster strong leadership within national and provincial health departments; building systems and processes for the efficient collection, and evidence-based use of data; building workforce skills in epidemiological and outbreak management and developing supply chain logistics.

Given the severely constrained capacity of many of the smaller, more remote and less well-developed PICTs to respond to COVID-19 it is encouraging to see that WHO is leading a regional approach to the response [23], including the development (not released at the time of writing) of a six-month *Pacific Action Plan for 2019 Novel Coronavirus (COVID-19) Preparedness and Response.* Central to this plan should be efforts to protect health systems and populations in less well-resourced PICT settings. This may include implementing key COVID-19 response activities, such as passenger screening and symptomatic case management at major regional transit hubs (i.e. Fiji in the south Pacific and Guam in the north Pacific) to prevent importation and risk of community transmission in less well-equipped states; drawing on regional resources, such as laboratories in Fiji, New Caledonia and French Polynesia, Guam and Hawai'i, that have advanced testing capabilities; and deployment of emergency medical teams, where necessary.

The results of this study should be interpreted with caution. Factors such as the impact of travel bans, change in passenger travel behaviours as a result of COVID-19, changes in the epidemiology of the virus, the potential for there to be unidentified cases or established community transmission in some settings and the impact of recent investments in public health surveillance and response are not considered in the tool. These limitations notwithstanding, the analysis provides a useful method to predict the more likely routes by which COVID-19 importation into the PICTs may occur and should be used to inform national and regional risk assessment activities. The tool may be of interest to those working in other settings.

As the global epidemiology of COVID-19 evolves, the results generated by this tool will need to be revised.

## Supplementary material

For supplementary material accompanying this paper visit http://dx.doi.org/10.1017/S0950268820000710. click here to view supplementary material
