# Supplementary material for: Risk of COVID-19 importation to the Pacific islands through global air travel
Source: Epidemiol Infect. 2020 Mar 23;148:e71. doi: 10.1017/S0950268820000710 (PMC7113321; doi:10.1017/S0950268820000710)
Supplement: Supplementary file 1 [file S0950268820000710sup001.pdf]

## Supplementary Table S1. Risk assessment table: risk of COVID-19 importation into the Pacific islands (as of 12 March 2020), by COVID-19 affected country.

| COVID-19 affected countries | COVID-19 cases | | Global Health Security Index | | Risk assessment | | | | | | | | | | | | | | |
|---|---|---|---|---|---|---|---|---|---|---|---|---|---|---|---|---|---|---|---|
| | | | | | North & South Pacific | | | North pacific | | | | | | South Pacific | | | | | |
| | | | | | | | | International travel to the North Pacific (per year) | | | | Risk score | Risk category | International travel to the South Pacific (per year) | | | | Risk score | Risk category |
| | Number of confirmed cases | Local transmission | Overall score (out of 100) | Global rank (of 195 countries) | (a) Number of cases | (b) Local transmission | (c) Level of preparedness | Up to 3 most frequented destinations* | Est. passenger volume | Est. percent of all travel to North Pacific from country | (d) Passenger volume | | | Up to 3 most frequented destinations* | Est. passenger volume | Est. percent of all travel to South Pacific from country | (d) Passenger volume | | |
| ***Western Pacific region*** | | | | | | | | | | | | | | | | | | | |
| China (inc. Hong Kong, Macau, & Taiwan) | 80,981 | Yes | 48.2 | 51 | 3 | 3 | 2 | CNMI | 171500 | 60% | 3 | 11 | H | PNG | 46000 | 38% | 2 | 10 | H |
| | | | | | | | | Guam | 65000 | 22% | 2 | 10 | H | Fiji | 40500 | 34% | 2 | 10 | H |
| | | | | | | | | Palau | 46000 | 16% | 2 | 10 | H | Samoa | 7500 | 6% | 1 | 9 | M |
| Republic of Korea | 7,869 | Yes | 70.2 | 9 | 3 | 3 | 1 | Guam | 667000 | 72% | 3 | 11 | H | Fiji | 11000 | 58% | 2 | 9 | M |
| | | | | | | | | CNMI | 233000 | 25% | 2 | 11 | H | N. Caledonia | 3500 | 18% | 1 | 8 | M |
| | | | | | | | | Palau | 22000 | 2% | 2 | 10 | H | PNG | 2000 | 10% | 1 | 8 | M |
| Japan | 620 | Yes | 59.8 | 21 | 3 | 3 | 2 | Guam | 538000 | 78% | 3 | 11 | H | N. Caledonia | 29500 | 30% | 2 | 10 | H |
| | | | | | | | | Palau | 72000 | 10% | 2 | 10 | H | Fiji | 26500 | 26% | 2 | 10 | H |
| | | | | | | | | CNMI | 60000 | 9% | 2 | 10 | H | F. Polynesia | 23000 | 23% | 2 | 10 | H |
| Singapore | 178 | Yes | 58.7 | 24 | 3 | 3 | 2 | Guam | 3500 | 73% | 1 | 9 | M | PNG | 32000 | 55% | 2 | 10 | H |
| | | | | | | | | - | - | - | - | - | - | Fiji | 13000 | 22% | 2 | 10 | H |
| | | | | | | | | - | - | - | - | - | - | Solomon Is | 6500 | 11% | 1 | 9 | M |
| Malaysia | 129 | Yes | 62.2 | 18 | 2 | 3 | 2 | - | - | - | - | - | - | PNG | 13000 | 68% | 2 | 9 | M |
| | | | | | | | | - | - | - | - | - | - | Fiji | 4000 | 20% | 1 | 8 | M |

| COVID-19 affected countries | COVID-19 cases | | Global Health Security Index | | Risk assessment | | | | | | | | | | | | | | |
|---|---|---|---|---|---|---|---|---|---|---|---|---|---|---|---|---|---|---|---|
| | | | | | North & South Pacific | | | North pacific | | | | | | South Pacific | | | | | |
| | | | | | | | | International travel to the North Pacific (per year) | | | | Risk score | Risk category | International travel to the South Pacific (per year) | | | | Risk score | Risk category |
| | Number of confirmed cases | Local transmission | Overall score (out of 100) | Global rank (of 195 countries) | (a) Number of cases | (b) Local transmission | (c) Level of preparedness | Up to 3 most frequented destinations* | Est. passenger volume | Est. percent of all travel to North Pacific from country | (d) Passenger volume | | | Up to 3 most frequented destinations* | Est. passenger volume | Est. percent of all travel to South Pacific from country | (d) Passenger volume | | |
| Australia | 122 | Yes | 75.5 | 4 | 2 | 3 | 1 | FSM | 3500 | 53% | 1 | 7 | M | Fiji | 401000 | 38% | 3 | 9 | M |
| | | | | | | | | Guam | 2000 | 34% | 1 | 7 | M | PNG | 250000 | 23% | 3 | 9 | M |
| | | | | | | | | - | | - | - | - | - | Samoa | 61500 | 9% | 2 | 8 | M |
| Philippines | 52 | Yes | 47.6 | 53 | 2 | 3 | 2 | Guam (58.6%) | 76000 | 59% | 2 | 9 | M | PNG | 47500 | 77% | 2 | 9 | M |
| | | | | | | | | CNMI | 34000 | 26% | 2 | 9 | M | Fiji | 5500 | 9% | 1 | 8 | M |
| | | | | | | | | FSM | 12000 | 9% | 2 | 9 | M | Solomon Is | 2500 | 4% | 1 | 8 | M |
| Viet Nam | 39 | Yes | 49.1 | 50 | 2 | 3 | 2 | Guam | 3000 | 91% | 1 | 8 | M | N. Caledonia | 1500 | 41% | 1 | 8 | M |
| New Zealand | 5 | Yes | 54 | 35 | 1 | 3 | 2 | - | - | - | - | - | - | Fiji | 261000 | 35% | 3 | 9 | M |
| | | | | | | | | - | - | - | - | - | - | Cook Is | 142000 | 19% | 3 | 9 | M |
| | | | | | | | | - | - | - | - | - | - | Samoa | 139000 | 19% | 3 | 9 | M |
| French Polynesia [territory] | 1 | No | NA | NA | 1 | 1 | 2 (est) | - | - | - | - | - | - | N. Caledonia | 17000 | 70% | 2 | 4 | L |
| | | | | | | | | - | - | - | - | - | - | Cook Is | 5000 | 19% | 1 | 3 | L |
| | | | | | | | | - | - | - | - | - | - | Fiji | 1500 | 5% | 1 | 3 | L |
| ***South-east Asia region*** | | | | | | | | | | | | | | | | | | | |
| India | 73 | Yes | 46.5 | 57 | 2 | 3 | 2 | - | - | - | - | - | - | Fiji | 8500 | 74% | 1 | 8 | M |
| | | | | | | | | - | - | - | - | - | - | PNG | 2000 | 17% | 1 | 8 | M |
| Thailand | 70 | Yes | 73.2 | 6 | 2 | 3 | 1 | Guam | 4000 | 79% | 1 | 7 | M | Fiji | 3000 | 30% | 1 | 7 | M |

| COVID-19 affected countries | COVID-19 cases | | Global Health Security Index | | Risk assessment | | | | | | | | | | | | | | |
|---|---|---|---|---|---|---|---|---|---|---|---|---|---|---|---|---|---|---|---|
| | | | | | North & South Pacific | | | North pacific | | | | | | South Pacific | | | | | |
| | | | | | | | | International travel to the North Pacific (per year) | | | | Risk score | Risk category | International travel to the South Pacific (per year) | | | | Risk score | Risk category |
| | Number of confirmed cases | Local transmission | Overall score (out of 100) | Global rank (of 195 countries) | (a) Number of cases | (b) Local transmission | (c) Level of preparedness | Up to 3 most frequented destinations* | Est. passenger volume | Est. percent of all travel to North Pacific from country | (d) Passenger volume | | | Up to 3 most frequented destinations* | Est. passenger volume | Est. percent of all travel to South Pacific from country | (d) Passenger volume | | |
| | | | | | | | | - | - | - | - | - | - | PNG | 3000 | 27% | 1 | 7 | M |
| | | | | | | | | - | - | - | - | - | - | N. Caledonia | 2000 | 19% | 1 | 7 | M |
| Indonesia | 34 | Yes | 56.6 | 30 | 2 | 3 | 2 | Guam | 5500 | 89% | 2 | 9 | M | PNG | 10000 | 45% | 2 | 9 | M |
| | | | | | | | | - | - | - | - | - | - | Fiji | 5500 | 25% | 1 | 8 | M |
| | | | | | | | | - | - | - | - | - | - | N. Caledonia | 2500 | 11% | 1 | 8 | M |
| Bangladesh | 3 | Yes | 35 | 113 | 1 | 3 | 2 | - | - | - | - | - | - | PNG | 1500 | 68% | 1 | 7 | M |
| Sri Lanka | 2 | No | 33.9 | 120 | 1 | 1 | 2 | - | - | - | - | - | - | Fiji | 1000 | 61% | 1 | 5 | L |
| ***Europe region*** | | | | | | | | | | | | | | | | | | | |
| Italy | 12,462 | Yes | 56.2 | 31 | 3 | 3 | 2 | - | - | - | - | - | - | F. Polynesia | 4000 | 67% | 1 | 9 | M |
| France | 2269 | Yes | 68.2 | 11 | 3 | 3 | 2 | - | - | - | - | - | - | F. Polynesia | 49500 | 53% | 2 | 10 | H |
| | | | | | | | | - | - | - | - | - | - | N. Caledonia | 40500 | 43% | 2 | 10 | H |
| | | | | | | | | - | - | - | - | - | - | Wallis & Futuna | 1000 | 1% | 1 | 9 | M |
| Spain | 2140 | Yes | 65.9 | 15 | 3 | 3 | 2 | - | - | - | - | - | - | F. Polynesia | 1500 | 56% | 1 | 9 | M |
| Germany | 1567 | Yes | 66 | 14 | 3 | 3 | 2 | - | - | - | - | - | - | F. Polynesia | 5000 | 50% | 1 | 9 | M |
| | | | | | | | | - | - | - | - | - | - | Fiji | 3000 | 30% | 1 | 9 | M |
| Switzerland | 645 | Yes | 67 | 13 | 3 | 1 | 2 | - | - | - | - | - | - | F. Polynesia | 2000 | 43% | 1 | 7 | M |
| | | | | | | | | - | - | - | - | - | - | N. Caledonia | 1500 | 30% | 1 | 7 | M |

| **COVID-19 affected countries** | **COVID-19 cases** | | **Global Health Security Index** | | **Risk assessment** | | | | | | | | | | | | | | |
|---|---|---|---|---|---|---|---|---|---|---|---|---|---|---|---|---|---|---|---|
| | | | | | **North & South Pacific** | | | **North pacific** | | | | | | **South Pacific** | | | | | |
| | | | | | | | | **International travel to the North Pacific (per year)** | | | | **Risk score** | **Risk category** | **International travel to the South Pacific (per year)** | | | | **Risk score** | **Risk category** |
| | **Number of confirmed cases** | **Local transmission** | **Overall score (out of 100)** | **Global rank (of 195 countries)** | **(a) Number of cases** | **(b) Local transmission** | **(c) Level of preparedness** | **Up to 3 most frequented destinations*** | **Est. passenger volume** | **Est. percent of all travel to North Pacific from country** | **(d) Passenger volume** | | | **Up to 3 most frequented destinations*** | **Est. passenger volume** | **Est. percent of all travel to South Pacific from country** | **(d) Passenger volume** | | |
| United Kingdom | 460 | Yes | 77.9 | 2 | 3 | 3 | 1 | - | - | - | - | - | - | Fiji | 7000 | 45% | 1 | 8 | M |
| | | | | | | | | - | - | - | - | - | - | F. Polynesia | 3500 | 22% | 1 | 8 | M |
| | | | | | | | | - | - | - | - | - | - | PNG | 1500 | 8% | 1 | 8 | M |
| Russian Federation | 20 | No | 44.3 | 63 | 2 | 1 | 2 | Guam | 5000 | 64% | 1 | 6 | L | - | - | - | - | - | - |
| | | | | | | | | CNMI | 3000 | 35% | 1 | 6 | L | - | - | - | - | - | - |
| ***Eastern Mediterranean region*** | | | | | | | | | | | | | | | | | | | |
| United Arab Emirates | 74 | Yes | 46.7 | 56 | 2 | 3 | 2 | - | - | - | - | - | | Fiji | 1500 | 59% | 1 | 8 | M |
| ***Americas region*** | | | | | | | | | | | | | | | | | | | |
| United States of America | 987 | Yes | 83.4 | 1 | 3 | 3 | 1 | Guam | 75500 | 54% | 2 | 9 | M | F. Polynesia | 169000 | 49% | 3 | 10 | H |
| | | | | | | | | Marshall Is | 19500 | 14% | 2 | 9 | M | Fiji | 67000 | 19% | 2 | 9 | M |
| | | | | | | | | CNMI | 16000 | 12% | 2 | 9 | M | A. Samoa | 31000 | 9% | 2 | 9 | M |
| Canada | 93 | Yes | 75.3 | 5 | 2 | 3 | 1 | - | - | - | - | - | - | Fiji | 12500 | 51% | 2 | 8 | M |
| | | | | | | | | - | - | - | - | - | - | F. Polynesia | 6500 | 27% | 1 | 7 | M |
| | | | | | | | | - | - | - | - | - | - | Cook Is | 3000 | 11% | 1 | 7 | M |
| Brazil | 52 | Yes | 59.7 | 22 | 2 | 3 | 2 | - | - | - | - | - | - | French Polynesia | 2000 | 87% | 1 | 8 | M |
| Chile | 23 | Yes | 58.3 | 27 | 2 | 3 | 2 | - | - | - | - | - | - | French Polynesia | 14000 | 94% | 2 | 9 | M |
| Argentina | 19 | No | 58.6 | 25 | 1 | 1 | 2 | - | - | - | - | - | - | French Polynesia | 2000 | 73% | 1 | 5 | L |

| COVID-19 affected countries | COVID-19 cases | | Global Health Security Index | | Risk assessment | | | | | | | | | | | | | | |
|---|---|---|---|---|---|---|---|---|---|---|---|---|---|---|---|---|---|---|---|
| | | | | | North & South Pacific | | | North pacific | | | | | | South Pacific | | | | | |
| | | | | | | | | International travel to the North Pacific (per year) | | | | Risk score | Risk category | International travel to the South Pacific (per year) | | | | Risk score | Risk category |
| | Number of confirmed cases | Local transmission | Overall score (out of 100) | Global rank (of 195 countries) | (a) Number of cases | (b) Local transmission | (c) Level of preparedness | Up to 3 most frequented destinations* | Est. passenger volume | Est. percent of all travel to North Pacific from country | (d) Passenger volume | | | Up to 3 most frequented destinations* | Est. passenger volume | Est. percent of all travel to South Pacific from country | (d) Passenger volume | | |

Number of cases: 1=<20; 2=10-150; 3=>150. Local transmission:1=no; 3=yes. Level of preparedness: 1=Global Health Security Index category 'most prepared'; 2= Global Health Security Index category 'very prepared'; 3=Global Health Security Index category 'least prepared'. Passenger volume (estimated volume of travel to north or south Pacific PICTs): 1=<10000; 2=10000-100000; 3=>100000. Risk score: Summation of component scores. Risk category: L=relatively lower risk (cumulative risk score of <7); M= relatively moderate risk (cumulative risk score of 7-9); H= relatively higher risk cumulative risk score of >9). Country and territory name abbreviations: A. Samoa = American Samoa; CNMI = Commonwealth of the Northern Mariana Islands; FSM = Federated States of Micronesia; F. Polynesia = French Polynesia; N. Caledonia = New Caledonia; PNG = Papua New Guinea.
